# Frontal lobe neurology and the creative mind

**DOI:** 10.3389/fpsyg.2014.00761

**Published:** 2014-07-23

**Authors:** Leonardo C. de Souza, Henrique C. Guimarães, Antônio L. Teixeira, Paulo Caramelli, Richard Levy, Bruno Dubois, Emmanuelle Volle

**Affiliations:** ^1^Neuropsychiatric Branch, Neurology Division, University Hospital, Universidade Federal de Minas GeraisBelo Horizonte, Brazil; ^2^Inserm, U 1127, ICM FrontlabParis, France; ^3^CNRS, UMR 7225, ICM FrontlabParis, France; ^4^Sorbonne Universités, UPMC Univ Paris 06, UMR S 1127Paris, France; ^5^Institut du Cerveau et de la Moelle épinière, ICM FrontlabParis, France; ^6^AP-HP, Hôpital Saint-Antoine, Service de NeurologieParis, France; ^7^AP-HP, Hôpital de la Salpétrière, Neurology Department, Institut de la Mémoire et de la Maladie d'AlzheimerParis, France

**Keywords:** creativity, prefrontal cortex, frontotemporal dementia, artistic, divergent thinking

## Abstract

Concepts from cognitive neuroscience strongly suggest that the prefrontal cortex (PFC) plays a crucial role in the cognitive functions necessary for creative thinking. Functional imaging studies have repeatedly demonstrated the involvement of PFC in creativity tasks. Patient studies have demonstrated that frontal damage due to focal lesions or neurodegenerative diseases are associated with impairments in various creativity tasks. However, against all odds, a series of clinical observations has reported the facilitation of artistic production in patients with neurodegenerative diseases affecting PFC, such as frontotemporal dementia (FTD). An exacerbation of creativity in frontal diseases would challenge neuroimaging findings in controls and patients, as well as the theoretical role of prefrontal functions in creativity processes. To explore this paradox, we reported the history of a FTD patient who exhibited the emergence of visual artistic productions during the course of the disease. The patient produced a large amount of drawings, which have been evaluated by a group of professional artists who were blind to the diagnosis. We also reviewed the published clinical cases reporting a change in the artistic abilities in patients with neurological diseases. We attempted to reconcile these clinical observations to previous experimental findings by addressing several questions raised by our review. For instance, to what extent can the cognitive, conative, and affective changes following frontal damage explain changes in artistic abilities? Does artistic exacerbation truly reflect increased creative capacities? These considerations could help to clarify the place of creativity—as it has been defined and explored by cognitive neuroscience—in artistic creation and may provide leads for future lesion studies.

Beyond its cultural, aesthetic or artistic aspects, creativity can be defined from a neuroscientific perspective as “the ability to produce a work that is both original (new, unusual, novel, unexpected) and valuable (useful, good, adaptive, appropriate)” (Sternberg and Lubart, 1999; Dietrich, 2004). Creative thinking usually involves the ability to break with conventional well-established ideas and to develop alternative behaviors in new and unexpected situations. In this sense, creativity may be considered to be a particular form of adaptation or problem solving (Runco, 2004; Sternberg, 2006). In this theoretical view, creativity relies on fundamental cognitive processes such as working memory, attention, planning, cognitive flexibility, mentalizing, and abstract thinking (Carlsson et al., 2000; Dietrich, 2004; Bogousslavsky, 2005; Changeux, 2005). These functions depend largely on the integrity of the prefrontal cortex (PFC), a brain region that is essential for behavioral adaptation and highly integrated mental functions. Functional neuroimaging data in healthy subjects also show that the PFC plays an important role in the cognitive processes involved in creativity (Gonen-Yaacovi et al., 2013). Therefore, both cognitive theories and neuroimaging data suggest that the integrity of the PFC is essential for creative thinking, and that neurological diseases that damage PFC regions (or their connections) would affect cognitive creativity processes. Some experimental studies have indeed demonstrated the impairment of creativity after prefrontal damage (Rankin et al., 2007; de Souza et al., 2010; Shamay-Tsoory et al., 2011; Abraham et al., 2012).

However, in contrast with these theories and experiments, a series of clinical observations reports the facilitation of artistic abilities in some patients with neurodegenerative disease affecting the frontal lobes, raising the question of a possible increased creativity following frontal damage (Palmiero et al., 2012; Schott, 2012; Gretton and ffytche, 2014). An exacerbation of creativity in neurological diseases affecting the frontal lobes would question the role of the PFC in creativity.

Herein, we propose that cognitive aspects of creativity depend on the integrity of PFC subregions and we hypothesize that some of these contradictory data may be reconciled by considering the repercussion of frontal symptoms into the patients' production, by taking into account affective and conative aspects of creativity, and by comparing the artistic and neuroscientific perspectives of creativity. This discussion will be illustrated using a clinical case of artistic production during the course of the behavioral variant frontotemporal dementia (bvFTD).

## Prefrontal functions and creativity

The PFC is highly developed in humans and plays a crucial role in elaborating and controlling voluntary and goal-directed behaviors, expanding behavior far beyond the sole repertoire of automatic and reflexive actions. The PFC enables adaptive behavior according to one's own objectives and to the context while taking into account past experiences and needs (Goldman-Rakic, 1995; Shallice and Burgess, 1996; Fuster et al., 2000; Miller and Cohen, 2001; Levy and Volle, 2009; Volle et al., 2013). This central role in adaptive behavior is supported by intense connections between the PFC and other brain regions (Dubois et al., 1995; Mesulam, 1998). The strong connective properties of this region suggest that the PFC is involved in integrating or combining different types of information according to the task goal. The PFC is connected with the sensory systems involved in perception, enabling access to information about the current environment. The PFC receives information about past events and knowledge though connections to long-term memory circuits. The PFC is also part of the limbic system and receives information on the individual needs, emotions, and motivations (Schoenbaum et al., 2009; Fellows, 2013) to guide decisions. The PFC interacts with motor systems that program, perform and monitor the plan of actions (Catani and Thiebaut de Schotten, 2012; Yeterian et al., 2012; Cole et al., 2013; Rojkova et al., under revision). Thus, the PFC can be considered to be a convergence hub that enables the integration of different types of information and the formation of mental representations of both the external and inner worlds (Ramnani and Owen, 2004; Reynolds et al., 2006; Nee et al., 2013) that can guide more sophisticated patterns of behavior.

Furthermore, the connections between the PFC and other brain regions are usually reciprocal, enabling the PFC to exert control over other brain systems, in addition to receiving information. For instance, control signals over the action system may inhibit actions that would not be suitable in a given context, and control over perceptual systems enables the selection of relevant information in the environment (Picton et al., 2007; Levy and Wagner, 2011; Volle et al., 2012). The supervisory role of the PFC also allows the selection and the voluntary retrieval of information in memory (Martin and Cheng, 2006; Thompson-Schill and Botvinick, 2006; Badre and Wagner, 2007; Strenziok et al., 2013). Several recent models describe a hierarchical postero-anterior organization of the control functions that are exerted by PFC in which an increased control requirement for behavioral adaptation recruits more anterior PFC subregions (Koechlin et al., 2003; Koechlin and Hyafil, 2007; Azuar et al., 2014). Other models also describe a posteroanterior PFC gradient in the abstraction degree of the mental representations that can be formed; more anterior regions support more abstract thinking (Christoff et al., 2001, 2009; Badre and Wagner, 2007; Volle et al., 2010).

Overall, the PFC enables the formation and control of mental representations according to an internal goal by selecting information from the environment or from memory, by forming or selecting rules, and by resisting spontaneous prepotent responses (Levy and Volle, 2009). These prefrontal properties are assumed to support creativity as well as complex human abilities such as planning, reasoning, problem solving, abstract thinking (Carlsson et al., 2000; Godefroy, 2003; Dietrich, 2004; Bogousslavsky, 2005; Changeux, 2005; Burgess et al., 2009; Levy and Volle, 2009). In other words, our knowledge of PFC structure and functions supports the assumption that the PFC is essential for cognitive processes that underlie creative thinking. Experimental studies using creativity tasks in healthy participants and in patients confirm this hypothesis.

## Experimental studies on the neural correlates of creativity

### Functional neuroimaging approach: a role for the FPC in creativity

Functional imaging studies have attempted to explore the cerebral bases of creativity processes using various experimental tasks (see Arden et al., 2010; Dietrich and Kanso, 2010; Jung, 2013 for reviews). Some studies relied on ecological tasks attempting to imitate creativity in real life, but most of them employed tasks drawn from theoretical cognitive models. Studies with a more ecological approach used tasks such as story writing (Bechtereva et al., 2004; Howard-Jones et al., 2005; Shah et al., 2013), object design (Kowatari et al., 2009; Ellamil et al., 2012), or music improvisation (Bengtsson et al., 2007; Berkowitz and Ansari, 2008; Limb and Braun, 2008; de Manzano and Ullen, 2012).

Among the studies based on theory-based creativity tasks, the most frequent framework used to examine the brain correlates of creativity was the divergent thinking approach (Runco and Acar, 2012). Divergent thinking tests typically require generating the maximal number of new or unusual responses. One of the classical divergent thinking tasks is the *Alternate Uses task*, which assesses the ability to produce many alternative uses of a common object such as a brick.

Another approach, which was proposed by Mednick (Mednick, 1962; Mednick et al., 1964), considers that creativity results from “the forming of associative elements into new combinations, which either meet specified requirements or are in some way useful. The more mutually remote the elements of the new combination, the more creative the process or solution.” One experimental task to test this hypothesis is to present three unrelated words without obvious connections between them (e.g., stain, glass, and red), and to ask the subject to find a fourth word that is related to each of these words (e.g., wine) (Jung-Beeman et al., 2004; Kounios et al., 2006). This task has been mainly used to investigate the phenomenon of “insight” or “Aha!” or “Eureka” (Kounios et al., 2006). “Aha” describes a subjective experience that occurs when solving a problem for which the solution suddenly comes to mind without effort or difficulty and is associated with a feeling of pleasure and confidence (Luo et al., 2004; Aziz-Zadeh et al., 2009; Qiu et al., 2010; Tian et al., 2011). This “Aha” experience is the cornerstone of another approach in creativity studies, that of problem solving with insight. Problems that raise an insight phenomenon include statements with strong implicit constraints that guide the search for a solution in the incorrect direction. The solution to these problems requires breaking these constraints and implicit associations and opening the search space to more possibilities. According to the classical model from Wallas (Kozbelt, 2011), this element is part of a creative process that follows four stages. Insight follows a preparation and an incubation phases and is followed by a verification phase. For many authors, the creative process is not this linear but instead alternates between phases of idea generation, evaluation, and the selection of ideas (Changeux, 2005; Simonton, 2010; Ward and Kolomyts, 2010; Ellamil et al., 2012).

A recent coordinate-based meta-analysis (Gonen-Yaacovi et al., 2013) using GingerALE free software (Eickhoff et al., 2012; http:www.brainmap.org/ale/) reviewed the published data regarding the investigation of the neural basis of creative thinking in functional neuroimaging studies. This study included 34 articles reporting 44 different experiments that employed the different creative paradigms aforementioned, i.e., divergent thinking tasks (Seger et al., 2000; Howard-Jones et al., 2005; Asari et al., 2008; Fink et al., 2009, 2010; Chrysikou and Thompson-Schill, 2011; Abraham et al., 2012; Ellamil et al., 2012; Kröger et al., 2012; Rutter et al., 2012) combination tasks and problem solving (Jung-Beeman et al., 2004; Luo et al., 2004; Geake and Hansen, 2005; Vartanian and Goel, 2005; Kounios et al., 2006; Mashal et al., 2007; Siebörger et al., 2007; Aziz-Zadeh et al., 2009; Qiu et al., 2010; Tian et al., 2011; Aziz-Zadeh et al., 2012; Cardillo et al., 2012; Green et al., 2012; Huang et al., 2013), as well as ecological tasks attempting to capture real life creativity instead of hypothesized cognitive processes (Bechtereva et al., 2004; Howard-Jones et al., 2005; Bengtsson et al., 2007; Berkowitz and Ansari, 2008; Limb and Braun, 2008; Kowatari et al., 2009; Ellamil et al., 2012; de Manzano and Ullen, 2012; Shah et al., 2013).

Despite the diversity of tasks used in these studies, the results showed a common set of brain regions as the neural basis of creative thinking, including multiple areas within the PFC and regions involved in semantic memory (the temporo-parietal region and posterior temporal and antero-lateral temporal cortex).

Additionally, this meta-analysis showed that distinct prefrontal subregions support distinct cognitive creativity processing. More specifically, tasks based on divergent thinking (to imagine alternative uses of objects or new designs) and those requiring the combination of information (to compose a sentence with unrelated words or to combine different figures to produce a new one, e.g.) were associated with both common and distinct prefrontal areas. Caudal lateral PFC was involved in both task categories, while more anterior PFC areas appear to be more task-oriented. For instance, within the frontal pole, the lateral part was more related to combination tasks, while its medial portion was engaged in divergent thinking tasks.

Together, these findings underlie the importance of PFC in creativity and suggest that different processes involved in creative thinking rely on distinct subregions within the PFC, in particular along the posterior-anterior axis and the medial-lateral axis. If PFC subregions are involved in creativity tasks, as suggested by functional imaging, one expects that damage to these areas would provoke impairment in the same tasks.

### Experimental patient studies: decreased creativity after prefrontal damage

Whether PFC regions are critical to creativity has been explored in very few patient studies. Creative thinking has been studied in patients with focal brain lesions (Shamay-Tsoory et al., 2011; Abraham et al., 2012) and in one of the most frequent causes of frontal damage: frontotemporal dementia (FTD) (Rankin et al., 2007; de Souza et al., 2010). FTD is a neurodegenerative disease and the second most common cause of dementia in patients under 65 years of age. FTD encompasses three different clinical syndromes: the behavioral variant (bvFTD) and the language variants, i.e., progressive non-fluent aphasia and semantic dementia (SD).

de Souza et al. (2010) investigated creativity in patients with bvFTD, using a standardized test of divergent thinking, the Torrance Test of Creative Thinking (TTCT; Torrance, 2004). The TTCT includes both verbal and figurative tasks. TTCT establishes objective criteria to measure creative production, by scoring three main aspects: (1) the fluency, i.e., the total number of responses, (2) the flexibility, i.e., the number of different categories to which the responses belong, and (3) originality, which is the number of new responses, here considered as responses that are statistically infrequent. Fluency and flexibility are usually defined as executive functions and are classically assessed in neuropsychological testing. The results from de Souza and colleagues showed that bvFTD patients performed worse than controls (a normal and a pathological control group) in all dimensions of the TTCT (fluency, flexibility, and originality) for both figurative and verbal tasks. bvFTD patients had also impaired performance in frontal functions such as flexibility, inhibition, abstraction and planning. These findings are consistent with previous data demonstrating that bvFTD patients have impairments in the production of new ideas either in an ecological task of artistic drawing or on the TTCT (Rankin et al., 2007). This study also showed that behavioral disorders such as perseverations and behavioral disinhibition (often sexual) could partly account for the “originality” of frontal patients in their responses in TTCT. In other words, some of the production features may be considered to be manifestations of the behavioral disorders that characterize bvFTD; these were not observed in the control subjects.

In this study, brain correlates of creative abilities were also explored in bvFTD patients, and perfusion in prefrontal regions measured using SPECT correlated with creativity performance at the TTCT (de Souza et al., 2010). More interestingly, there was a clear concordance among the regions reported in this study and those observed in functional neuroimaging studies in healthy subjects (Gonen-Yaacovi et al., 2013), in particular in the left inferior frontal gyrus [BA 47], the left posterior inferior and middle temporal gyri [BA 37], the left inferior parietal lobule [BA39/40], and the left precuneus [BA 23].

Focal prefrontal lesions also impact creative thinking, as demonstrated by two recent lesion studies that examined the consequences of focal brain damage (such as stroke) on creative performance (Shamay-Tsoory et al., 2011; Abraham et al., 2012). Shamay-Tsoory et al. (2011) compared patients' performance on the TTCT according to distinct lesion locations: frontal pole, posterior part of the PFC, or outside the PFC. The results showed that damage to the frontal pole was specifically associated with a deficit at the TTCT. More especially, the originality criterion was the most compromised, and patients with damage to the frontal pole were less original in their response than other patients. Abraham et al. (2012) used several creativity tests in patients with various lesion locations and showed that patients with lateral frontal damage were impaired in both fluency and originality aspects of divergent thinking tasks.

Taken together, these data supports the critical role of PFC in creative thinking. From a cognitive perspective, cerebral findings from patient studies agree with functional neuroimaging results (Carlsson et al., 2000; Seger et al., 2000; Bechtereva et al., 2004; Jung-Beeman et al., 2004; Goel and Vartanian, 2005; Howard-Jones et al., 2005; Asari et al., 2008; Aziz-Zadeh et al., 2009; Fink et al., 2009, 2010; Kowatari et al., 2009). These findings are also consistent with studies that used SPECT (Chavez-Eakle et al., 2007), voxel-based morphometry (Jung et al., 2010b; Takeuchi et al., 2010a; Gansler et al., 2011), and diffusion tensor imaging (Jung et al., 2010a; Takeuchi et al., 2010b).

However, against all odds, a series of medical observations have reported the facilitation of artistic abilities in patients with damage to the frontal lobes (Palmiero et al., 2012; Schott, 2012).

## Clinical observations of creativity in neurological patients

The description of patients developing artistic abilities raises the question of enhanced creativity following frontal damage, which would challenge the neuroimaging findings in controls and patients and the theoretical role of prefrontal functions in creativity processing. To better understand the relationships between frontal damage, frontal functions, artistic ability, and creativity, we performed a mini-review of published articles reporting changes in artistic production by neurological patients.

### A mini-review of medical reports on creativity

We actively searched the *PubMed database* for previous medical reports of changes in artistic skills in neurological patients. Unlike experimental studies on creativity that were usually based on various experimental tasks using objective measures and more instructed tasks, these clinical reports were based on a subjective evaluation of spontaneous patients' productions in the artistic domain. We used the following key-words terms: “dementia, frontotemporal+dementia, Alzheimer's+disease, semantic+dementia, or stroke” AND “creativity, artistry, or artist.” We looked for articles published until March 2014. We also included articles cited in previous reviews on creativity in patients (Palmiero et al., 2012; Schott, 2012; Gretton and ffytche, 2014). We did not include Parkinson disease, as artistic facilitation in this condition may most likely relate to the dopa medication rather than to the brain damage itself (Lhommee et al., 2014). The papers found throughout this research were evaluated for relevance and duplicate cases were excluded.

We found 35 relevant papers reporting the degradation, emergence, preservation or improvement of creative expression in 53 patients after the onset of different neurological diseases (see Table 1): 19 patients with temporal variant FTD (semantic dementia), 10 patients with behavioral variant FTD, eight patients with Alzheimer's disease, four patients with primary progressive non-fluent aphasia, and 12 patients with various neurological diseases (Espinel, 1996; Miller et al., 1998, 2000; Crutch et al., 2001; Thomas-Anterion et al., 2002, 2010; Kleiner-Fisman et al., 2003; Mell et al., 2003; Mendez and Perryman, 2003; Annoni et al., 2005; Fornazzari, 2005; Lythgoe et al., 2005; Serrano et al., 2005; Chatterjee et al., 2006; Drago et al., 2006a,b; Budrys et al., 2007; Finney and Heilman, 2007; Midorikawa et al., 2008; Seeley et al., 2008; Liu et al., 2009; Thomas-Anterion, 2009; Chakravarty, 2011; Chatterjee et al., 2011; van Buren et al., 2013; Galarza et al., 2014; Takahata et al., 2014). All reported patients with temporal FTD (*n* = 19) presented the emergence (*n* = 11), increase (*n* = 2), or preservation (*n* = 6) of creative production but no degradation of artistic abilities (Miller et al., 1996, 1998; Edwards-Lee et al., 1997; Drago et al., 2006b; Wu et al., 2013). Most case reports on behavioral variant FTD (*n* = 10) noted the emergence (*n* = 4), increase (*n* = 4), or preservation (*n* = 1) of artistic abilities (Miller et al., 1998; Thomas-Anterion et al., 2002; Mendez and Perryman, 2003; Serrano et al., 2005; Liu et al., 2009; Thomas-Anterion, 2009). The effects of Alzheimer's disease on artistic production were more heterogeneous, with observations of both increase (Fornazzari, 2005; Chakravarty, 2011) and degradation (Cummings and Zarit, 1987; Crutch et al., 2001; Serrano et al., 2005; van Buren et al., 2013). Other neurological degenerative diseases or strokes of various locations were associated with heterogeneous profiles (Annoni et al., 2005; Lythgoe et al., 2005; Thomas-Anterion et al., 2010; Takahata et al., 2014). The cognitive, behavioral, and artistic changes reported in the reviewed studies are synthetized in Table 2.

**Table 1:** **Synthesis of published articles reporting changes in artistic creativity in neurological patients**.

**Author and year**	**Diagnosis**	**Neuroimaging data**	**Change in abilities**	**Previous interest in Art?**	**Art domain**	**Neuropsychological data**
Miller et al., 1998, 2000	Frontal FTD	SPECT: bifrontal and temporal hypoperfusion (right > left)	E	Occasionally produced novels (not a professional)	Photo	MMSE = 26/30
(Pt 3)						Preserved language and constructions
						Impaired executive tests (WCST, Stroop, TMT)
						Behavioral disinhibition and compulsions
Thomas-Anterion et al., 2002 and Thomas-Anterion, 2009	Frontal FTD	CT scan: frontotemporal atrophy	E	No	Drawing	Language and memory impairment
		SPECT: frontal hypoperfusion				Impaired executive tests
						Emotional difficulties
						Apathy
						Stereotypies
Mendez and Perryman, 2003	Frontal FTD	MRI: frontotemporalatrophy	I	Yes (professional graphic artist)	Drawing	MMSE = 22/30
(Pt 1)		PET-FDG: Bifrontal and right temporal hypometabolism				Preserved language, face processing and visuospatial tests
						Decreased verbal fluency
						Concrete interpretation of proverbs
						Compulsions and hoarding
						Poor insight
Mendez and Perryman, 2003	Frontal FTD	MRI: normal	I	Yes (professional photographer and graphic designer)	Drawing	MMSE = 23/30
(Pt 2)		SPECT: Bifrontal and right temporal hypoperfusion				Preserved visuospatial and face processing tests
						Decreased verbal fluency, executive functions and memory
						Difficulties with proverbs
						Inappropriate social behaviors and compulsions
						Loss of insight
Mendez and Perryman, 2003	Frontal FTD	MRI: frontotemporal atrophy	I	Occasionally caricatures (not a professional)	Drawing	MMSE 20/30
(Pt 3)		SPECT: Frontal and right anterior temporal hypoperfusion				Preserved visuospatial and face processing tests
						Decreased verbal fluency and memory
						Difficulties with similarities and proverbs
						Poor insight
						Compulsions
						Disinhibited behaviors, impulsivity
Mendez and Perryman, 2003	Frontal FTD	MRI: frontotemporal atrophy	P	Yes (professional artist)	Not specified	MMSE 23/30
(Pt 4)		SPECT: Bifrontal and bitemporal hypoperfusion				Preserved visuospatial and face processing tests
						Decreased verbal fluency
						Good proverb interpretation
						Disinhibition of personal behavior
						Compulsive behaviors
Serrano et al., 2005	Frontal FTD	MRI: normal	I	Yes (painter)	Painting	Impaired language skills
(Pt 3)		SPECT: Left fronto-temporoparietal hypoperfusion				Impaired executive tests (TMT, spans)
						Preserved performance on similarity test
						Compulsive behaviors
Liu et al., 2009	Frontal FTD (a)	MRI: atrophy in bilateral anterior and left lateral frontal regions.	E	No	Painting Sculpture	MMSE 28/30
						Preserved visuospatial skills
						Impaired executive tests
						Abstraction difficulties
						Lack of emotion, empathy and insight
						Impaired verbal memory and semantic
						Antisocial and compulsive behaviors
						Paintings contain sexual disinhibition
						Obsessions about art and dots and stripes
Thomas-Anterion, 2009	Frontal FTD	No imaging data	E	No	Drawing	No neuropsychological data
(Pt 2)					Poetry	Obsession about art
Budrys et al., 2007	Frontal FTD (b)	MRI: bilateral frontotemporal atrophy	D	Yes (professional artist)	Painting	MMSE 25/30
						Aphasia and amnesia
						Difficulties on abstract reasoning
						Verbal and writing perseverations
Edwards-Lee et al., 1997	Temporal FTD	MRI: bitemporal atrophy, SPECT: Bitemporal hypoperfusion	P	Yes (pianist)	Music	MMSE = 1/30
(Pt LTLV 1) and Miller et al., 2000						Preserved attentional and visuospatial skills
						Impaired executive tests (Stroop, TMT)
						Compulsive behaviors
Edwards-Lee et al., 1997	Temporal FTD	MRI: left temporal lobe atrophy	P	Yes	“Artistic skills”	MMSE 26/30
(Pt LTLV 3) and Miller et al., 2000		SPECT: Left temporal hypoperfusion				Preserved visuospatial skills
						Semantic anomia
						Memory impairment
Edwards-Lee et al., 1997	Temporal FTD	MRI: generalized atrophy	E	No	Painting	MMSE = 15/30
(Pt LTLV 5) and Miller et al., 2000		SPECT: Bitemporal hypoperfusion				Preserved visuospatial skills
						Executive tests markedly impaired (TMT, Stroop, verbal fluency)
						Anomic aphasia and impaired memory
Miller et al., 1998 and 2000	Temporal FTD (c)	SPECT: bitemporal (Left > right) and mild left frontal hypoperfusion	E	No	Painting drawing	MMSE = 16/30
						Preserved visuospatial skills
						Letter fluency = 2
						Perseverations on executive tests
						Disinhibition and compulsive behavior
Miller et al., 1998	Temporal FTD	No imaging data	E	No	Painting	No neuropsychological data Disinhibition in language.
Miller et al., 1998 and 2000	Temporal FTD	MRI: bifrontal and left temporal atrophy	I	Yes	Sculpture	MMSE = 9/30
		SPECT: Left frontal and bitemporal hypoperfusion				Mild deficit in visuospatial tests
						Decreased verbal fluency
						Impaired memory and naming
						Disinhibition and compulsive behavior
Miller et al., 1998 and 1998 (also in Miller et al., 1996 and Edwards-Lee et al., 1997 Patient RTLV 4)	Temporal FTD	MRI: bitemporal atrophy	E	No	Painting	MMSE = 15/30
		SPECT: Bilateral temporal hypoperfusion				Fluent verbal output, with semantic anomia
						Letter fluency = 2
						Disinhibition and compulsive behavior
Midorikawa et al., 2008	Temporal FTD	MRI: left temporal atrophy	E	No	Painting	Language deficits (semantic deficits)
(Pt 1)						Abnormal behaviors (intrusiveness, repetitive actions)
Midorikawa et al., 2008	Temporal FTD	MRI: left temporal atrophy	E	No	Painting	Language deficits (semantic deficits)
(Pt 2)						
Miller et al., 2000	Temporal FTD	SPECT: bitemporal, left greater than right, hypoperfusion with frontal sparing	P	Yes (previous inventor)	Inventor	MMSE = 21/30
(Pt 1)						Boston naming test: 1/60
						Normal on Rey Complex Figure
						Disinhibited behavior
Miller et al., 2000	Temporal FTD	MRI: focal left temporal atrophy	P	Yes (previous bridge)	Bridge	MMSE = 25/30
(Pt 2)		SPECT: bitemporal, left greater than right, hypoperfusion with frontal sparing				Normal on Wisconsin Card Sort Test
						Normal visual reproduction abilities
						Intact social skills
Miller et al., 2000	Temporal FTD	SPECT: bitemporal hypoperfusion with frontal sparing	P	Yes (previous inventor)	Inventor	MMSE = 22/30
(Pt 3)						Boston naming test: 16/60
						Apathy
Miller et al., 2000 (Pt 4)	Temporal FTD	SPECT: bitemporal, left greater than right, hypoperfusion with frontal sparing	E	No	Music	MMSE = 17/30
						Boston naming test: 4/60
						Normal visual reproduction abilities
						Personality changes (childlike, euphoric)
						Compulsive behavior
Miller et al., 2000	Temporal FTD	SPECT: moderate left temporal and mild left frontal hypoperfusion	E	No	Music	MMSE = 25/30
(Pt 5)						Decreased verbal output
Miller et al., 2000	Temporal FTD	Positron emission tomography showed left anterior hypometabolism	P	Yes (music)	Music	MMSE = 15/30
(Pt 6)						Fluent speech with perseverations
Drago et al., 2006a	Temporal FTD	MRI: anterior bitemporal atrophy	I	Yes (visual artist)	Painting	Preserved visuospatial skills
						Language deficits
						Behavioral disorders (more impulsive and belligerent)
Wu et al., 2013	Temporal FTD	MRI: bilateral (left greater than right) anterior temporal atrophy extending to hippocampal and orbitofrontal regions	E	No	Verbal (poetry)	MMSE 26/30
(Pt 1)						Normal performance on visual memory and visuospatial function
						Impairment in verbal memory
						Preserved executive function
						Disinhibition
Wu et al., 2013	Temporal FTD	MRI: atrophy in (left greater than right) anterior temporal lobe atrophy	E	No	Verbal (rhyming)	MMSE 30/30
(Pt 2)						Marked anomia, with intact comprehension and repetition
						Impairment in executive functions and in visual memory
						Preserved short-term verbal memory
Wu et al., 2013	Temporal FTD	MRI: marked atrophy in the anterior temporal lobes and amygdala, right greater than left, with moderate atrophy of the orbitofrontal cortex, right anterior insula, and right parahippocampus	E	No	Verbal (writer)	MMSE 28/30
(Pt 3)						Poor performance on tasks of semantic knowledge, executive function and famous face recognition
						Disinhibition
Mell et al., 2003	PPA	MRI: bifrontal atrophy and mild temporal atrophy	I	Yes (art teacher)	Painting	Preserved visuospatial skills
	(Non-Fluent)					Non-fluent and effortful language
						Impaired executive tests
Serrano et al., 2005	PPA	CT scan: diffuse cortical atrophy with left predominance	P	Yes (painter)	Painting	Preserved visuospatial skills
(Pt 2)	(Non-Fluent)					Language deficits
Finney and Heilman, 2007	PPA	MRI: focal atrophy of the left anterior temporal lobe and left insula	D	Yes (painter)	Painting	MMSE 25/30
	(Non-Fluent)					Boston naming test 47/60
						Categorical letter fluency 8
						Preserved visuospatial skills
Seeley et al., 2008	PPA (d)	MRI: predominantly left inferolateral frontal atrophy	I	Yes	Visual Art	MMSE = 20/30
		SPECT: Predominantly left frontal hypoperfusion				Deficits limited to language and executive functions (span; fluency; TMT); Perseverations
Espinel, 1996	Mixed Alzheimer's disease	No imaging data	I	Yes (professional artist)	Painting	No neuropsychological data
Cummings and Zarit, 1987	Alzheimer's disease	No imaging data	D	Yes (professional artist)	Painting	MMSE: varies from 21 to 10 over 2.5 years
						Boston naming test: varies from 28 to 19 over 2.5 years
						FAS: varies from 7 to 0 over 2.5 years
Crutch et al., 2001 (and van Buren et al., 2013, Pt 1)	Alzheimer's disease	MRI: generalized brain atrophy	D	Yes (professional artist)	Painting	MMSE 22/30
					Drawing	WAIS 94
						Calculation 0/24
						Impaired visuospatial abilities
						Impaired verbal memory
Maurer and Prvulovic, 2004	Alzheimer's disease	No imaging data	D	Yes (professional artist)	Painting	Severe visuoconstructive deficits
					Drawing	Prosopagnosia
						Gestural apraxia
Fornazzari, 2005	Alzheimer's disease	MRI: large arachnoid cyst	P	Yes (painter)	Painting	MMSE 26/30
		SPECT: Bilateral temporo-parietal hypoperfusion				Preserved visuospatial abilities
						Deficits in episodic memory, language, gestural praxis and executive functions
Serrano et al., 2005	Alzheimer's disease	CT scan: diffuse cortical atrophy	D	Yes (painter)	Painting	MMSE = 22/30
(Pt 1)						Impaired visuospatial skills
						Impaired executive tests (similarities, TMT) and memory
Chakravarty, 2011	Alzheimer's disease	CT scan: Diffuse cortical atrophy	E	No	Painting	MMSE = 16/30
						CDR = 3
van Buren et al., 2013	Alzheimer's disease	No imaging data	D	No	Painting	Short term memory loss and emotional dysregulation
(Pt 2)						Memory impairment
Kleiner-Fisman et al., 2003	Corticobasal degeneration	MRI: right-predominant atrophy	D	Yes (professional illustrator)	Graphic	Severely impaired visuo-spatial skills, spatial neglect
		PET-FDG: marked hypoperfusion on right hemisphere and left frontal region			Arts	Deficits on attention, initiation, memory and executive functions
						Poor insight
						Personality changes, irritability
						Apathy
Sahlas, 2003	Lewy Body	No imaging data	D	Yes (professional artist)	Painting	No neuropsychological data but reports of deterioration of visuospatial functions
	Dementia				Writing	
Drago et al., 2006a	Lewy Body	No imaging data	D	Yes (visual artist)	Painting	MMSE = 6/30
	Dementia					Poor orientation and apraxic gaze
Annoni et al., 2005	Stroke	MRI: left occipital region (V1 and V2)	I	Yes (professional painter)	Painting	MMSE = 29/30
(Pt 1)						Normal neuropsychological exam
						Emotional dysfunction
						Increased impulsiveness
Annoni et al., 2005	Stroke	MRI: left paramedian thalamus infarct	I	Yes (professional painter)	Painting	MMSE = 28/30
(Pt 2)						Normal neuropsychological exam
						Mild emotional dyscontrol
						Moderate tendency to perseveration in phonological and figural fluency
						No compulsive behaviors
Chatterjee et al., 2011	Stroke	No imaging data (left hemisphere stroke)	Change	Yes (professional painter)	Painting	No neuropsychological data
(Pt 1)						
Chatterjee et al., 2011	Stroke	No imaging data (left hemisphere stroke)	Change	Yes (professional painter)	Painting	No neuropsychological data
(Pt 2)						
Chatterjee et al., 2011	Stroke	No imaging data (right hemisphere stroke)	Change	Yes (professional painter)	Painting	No neuropsychological data but reports left spatial neglect
(Pt 3)						
Takahata et al., 2014	Stroke	CT and MRI: infarction in the left prefrontal region	I	Yes	Painting	MMSE = 26/30
						Preserved visuospatial abilities
						Deficits in episodic memory and executive functions
						Behavioral perseverations
						No impulsiveness and no compulsiveness.
Thomas-Anterion, 2009	Stroke	MRI: left posterior insula and parietal operculum infarct	E	No	Painting	Normal neuropsychological exam
(Pt 3)						Compulsive art production with specific colors
Lythgoe et al., 2005	Subarachnoid hemorrhage	CT: no focal injury	E	No	Painting	Almost normal, except difficulties in switching and inhibition control
					Poetry	Patient obsessed with art
					Sculpture	
Galarza et al., 2014	Intracerebral hemorrhage associated to a cerebral arteriovenous malformation	MRI: extensive damage of left temporal lobe due to lobectomy.	Change	Yes	Music	Low performance in confrontation naming tests. Impairment on episodic memory tests for verbal modality, but not for visual modality. Preserved emotion recognition, except for fear.

*This table synthesizes the published medical reports of changes in artistic skills in neurological patients. Abbreviations: CT, Computerized tomography; D, Degradation of artistic abilities; E, Emergence of artistic abilities; FTD, Frontotemporal dementia; I, Increase of artistic abilities; MMSE, Mini-Mental State Examination; P, Preservation of preceding artistic abilities; PET-FDG, Fluorodeoxyglucose positron emission tomography; PPA, Primary Progressive Aphasia; Pt, Patient; SPECT, Brain perfusion scintigraphy; TMT, Trail Making Test; WCST, Wisconsin Card Sorting Test. (a) Frontal FTD associated to ALS in a patient with previous bipolar disorder; (b) Frontal FTD due to Neuronal Intermediate Filament Inclusion Disease; (c) Temporal FTD associated to ALS; (d) Primary Progressive Aphasia due to corticobasal degeneration*.

**Table 2:** **Synthesis of cognitive, behavioral and artistic changes in previous published cases of patients listed in Table 1 and in our patient**.

**Pathology**	**Bv-FTLD**	**Temp-FTLD**	**nfPPA-FTLD**	**Alzheimer's disease**	**Other**
Number of patients	11	19	4	8	12
Artistic emergence	5	11	0	1	2
Artistic increase (or preservation)	4 (1)	2 (6)	2 (1)	1 (1)	4 (3)
Artistic degradation	1	0	1	5	3
Artistic domain = visual	10	8	4	8	11
Intact visuospatial abilities	7 out of 7 reported	9 out of 10 reported	3	The degradation of artistic skills was associated with impaired visuospatial abilities in 6 cases out of 8 reported	
Positive behavioral symptoms reported					
Perseverations	3	2	1	–	1
Disinhibition	5	7	–	–	2
Compulsions obsessions	9	5	–	–	2
Negative dysexecutive symptoms reported					
1 or several deficits	10 out of 10 reported	7 out of 9 reported	3	3	3
Abstraction difficulties	5 out of 8 reported	–	–	1	–

*This table summarizes the patterns of artistic changes (emergence, increase/preservation, or degradation of artistic abilities) and behavioral and neuropsychological findings in previously reported neurological patients. Neuropsychological deficits and behavioral disorders may be underreported due to the absence of specific mention in the original papers. “out of x reported” means the number of patients for which this given cognitive or behavioral aspect was assessed and reported in the article. We did not include Parkinson's disease because changes in creativity in these patients may be linked with dopamine rather than neurodegeneration. Bv-FTD, Behavioral variant of fronto-temporal lobar degeneration; temp-FTD, temporal variant of fronto-temporal lobar degeneration or semantic dementia; PPA-FTD, non-fluent primary progressive aphasia form of fronto-temporal lobar degeneration; Other, Corticobasal degeneration, Lewy body dementia, stroke, subarachnoid hemorrhage and cerebral arteriovenous malformation*.

This non-systematic review highlights that some FTD patients develop enhanced artistic abilities and suggests that the relations between FTD, frontal functions, artistic abilities and creativity are unclear, as discussed below. We first would like to illustrate the paradoxical relationship between frontal symptoms and creativity by reporting the clinical observation of a patient who developed artistic abilities during the course of bvFTD. This is a new clinical case (unpublished original data) that will be discussed in conjunction with the other reviewed findings.

### Clinical vignette

Mrs. YCFZ (case number 963564), a retired dentist secretary aged 83 years, was evaluated in October 2010 in the Cognitive and Behavioral Neurology Unit of the Clinics Hospital from the Federal University of Minas Gerais (Belo Horizonte, Brazil). She was referred to the unit for the evaluation of behavioral and cognitive symptoms that had been evolving for approximately 2 years. Her preceding medical history was unremarkable, except for systemic hypertension, which was well controlled.

The family reported that the patient demonstrated striking behavioral changes. She was progressively uninterested in previously appreciated household chores, and she narrowed her usual cooking repertoire, abandoning the preparation of traditional dishes from her native country, El Salvador. Increased appetite manifested as a troublesome binge eating cookies. Additionally, the patient became progressively less concerned with personal grooming. The patient developed a new stereotyped and fixed routine. For example, she started to eat one banana every day at 10 o'clock precisely. She also presented with repetitive and ritualistic behaviors such as compulsive writing, obsessions regarding time schedules and compulsive handbag checking. In this context, the patient started to produce drawings in a compulsive manner. Mrs. YCFZ also had memory complaints, but behavioral disorders remained the most impaired domain throughout the course of the illness. Basic activities of daily living were globally preserved, although she needed assistance for some instrumental activities such as financial operations.

**Figure 1:**
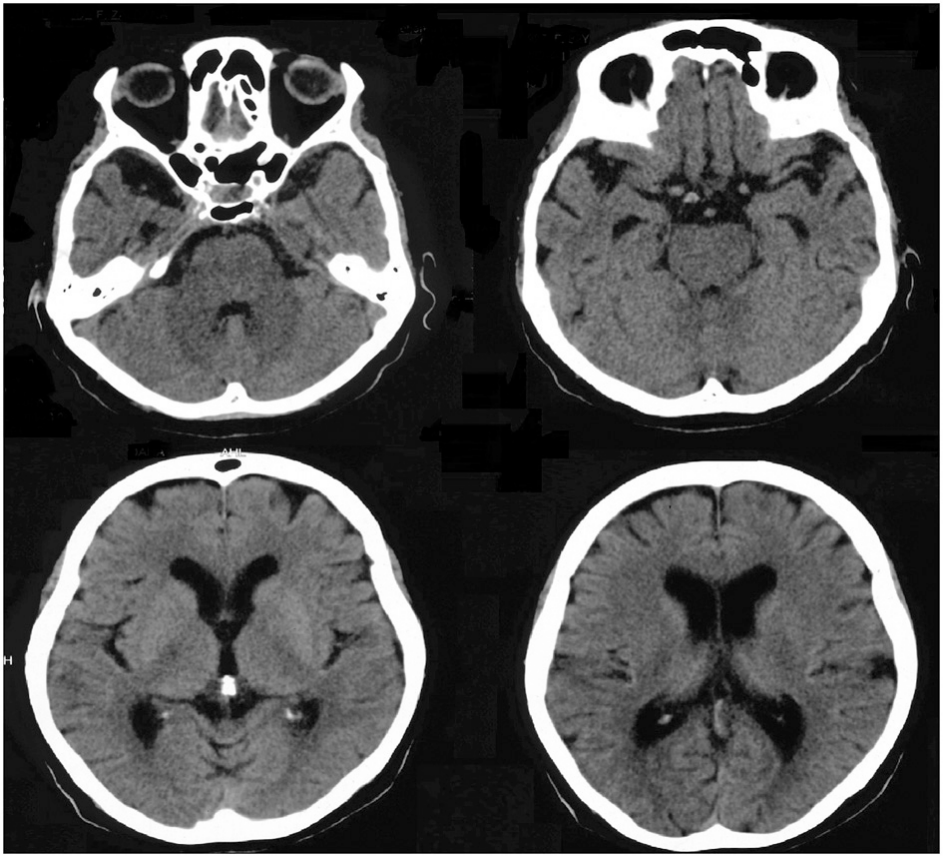
**Brain computed tomography scan performed in 2009 showing marked atrophy in bilateral temporopolar and frontal regions**.

The standard neurological examination was normal, without eye movement disorders or extrapyramidal signs. Formal neuropsychological evaluation (November 2010—Table 3) showed an impairment in global cognitive efficiency both on the Mini-Mental State Exam (MMSE: 16/30; Folstein et al., 1975) and on the Mattis Dementia Rating Scale (103/144; Porto et al., 2003). Executive tasks such as DRS initiation/perseveration subscale, FAS letter fluency and digit span were altered. There was a marked episodic memory deficit, which was characterized by low performance on both learning and delayed recall tasks from the Rey Auditory Verbal Learning Test (Malloy-Diniz et al., 2007) and in the DRS memory subscale. There was a moderate impairment in the naming task (9/15; Bertolucci et al., 2001). The visual abilities assessed using the Visual Object and Space Perception Battery (Warrington and James, 1991; Quental et al., 2013) were preserved (number location and cube analysis). The patient had no deficit on gesture execution, and no signs of Balint or Gertsmann syndromes. Brain computed tomography scanning in 2009 showed a remarkable atrophy in temporopolar regions bilaterally and a mild frontal polar atrophy (Figure 1). Brain MRI performed 2 years later showed no signs of cerebrovascular disease and confirmed the same regional atrophy pattern with additional diffuse brain shrinkage. On clinical follow-up after 36 months, the global cognitive efficiency assessed using MMSE remained stable (see Table 4), although language and functional abilities deteriorated, as assessed using the Functional Activities Questionnaire (Pfeffer et al., 1982). The diagnosis of probable bvFTD was retained on a clinical basis.

**Table 3:** **Neuropsychological assessment of the patient YCFZ (November 2010)**.

**Test**	**Patient score**	**Standard deviation**
**MATTIS TOTAL SCORE (/144)**	**103**	−**9.47**
*MATTIS–Attention (/37)*	33	−1.83
*MATTIS–Initiation (/37)*	19	−4.26
*MATTIS–Construction (/6)*	6	
*MATTIS–Concepts (/39)*	37	
*MATTIS–Memory (/25)*	8	−2.59
**Verbal SPAN (DIRECT–INDIRECT)**	**4–3**	
Rey auditory verbal learning test		
*Immediate recall list A*	18	−6.1
*Delayed recall list A*	0	−2.6
*Recognition test list A*	3	−4.3
**NAMING (BOSTON–CERAD) (/15)**	**9**	
FAS–Total	19	−9.9
Letter F	7	
Letter A	6	
Letter S	6	
**VISUAL OBJECT AND SPACE PERCEPTION**		
*Number location (/20)*	20	Cut-off: 9^*^
*Cube analysis (/20)*	20	Cut-off: 9^§^

* *This cut-off distinguished controls from patients with early Alzheimer's disease with 63% sensitivity and 74% specificity (Quental et al., 2013)*.

§ *This cut-off distinguished controls from patients with early Alzheimer's disease with 75% sensitivity and 68% specificity (Quental et al., 2013)*.

**Table 4:** **Longitudinal cognitive assessment of Mrs YCFZ, from November 2010–September 2013**.

	**November 2010**	**January 2011**	**May 2011**	**February 2012**	**June 2012**	**September 2012**	**November 2012**	**February 2013**	**September 2013**
Time orientation (/5)	2	1	1	1	0	1	1	0	0
Spatial orientation (/5)	4	4	3	4	3	2	3	3	3
Registration (/3)	2	3	3	3	3	3	3	3	3
Mental calculation (/5)	0	1	1	0	1	1	0	0	2
Recall (/3)	0	0	0	0	0	0	1	0	0
Language (/8)	7	8	8	8	8	8	8	8	8
Copy (/1)	1	1	1	0	1	0	0	1	1
**MMSE (/30)**	**16**	**18**	**17**	**16**	**16**	**15**	**16**	**15**	**17**
Animal Fluency (Cut-off: 13)	9	7	5	5	6	NA	6	8	7
Functional Activities Questionnaire (0–30)	23	NA	NA	29	26	29	30	28	30

*The table presents the MMSE total scores (in bold) and subscores for time and spatial orientation, registration of three words, mental calculation, recall of three words, language and copy of pentagons. Data for Animal Fluency and for the Functional Activities Questionnaire–FAQ (Pfeffer et al., 1982) for Activities of Daily Living are also presented. A cut-off point higher than 9 in the FAQ indicates impaired function and cognitive impairment. (NA, Not available)*.

**Figure 2:**
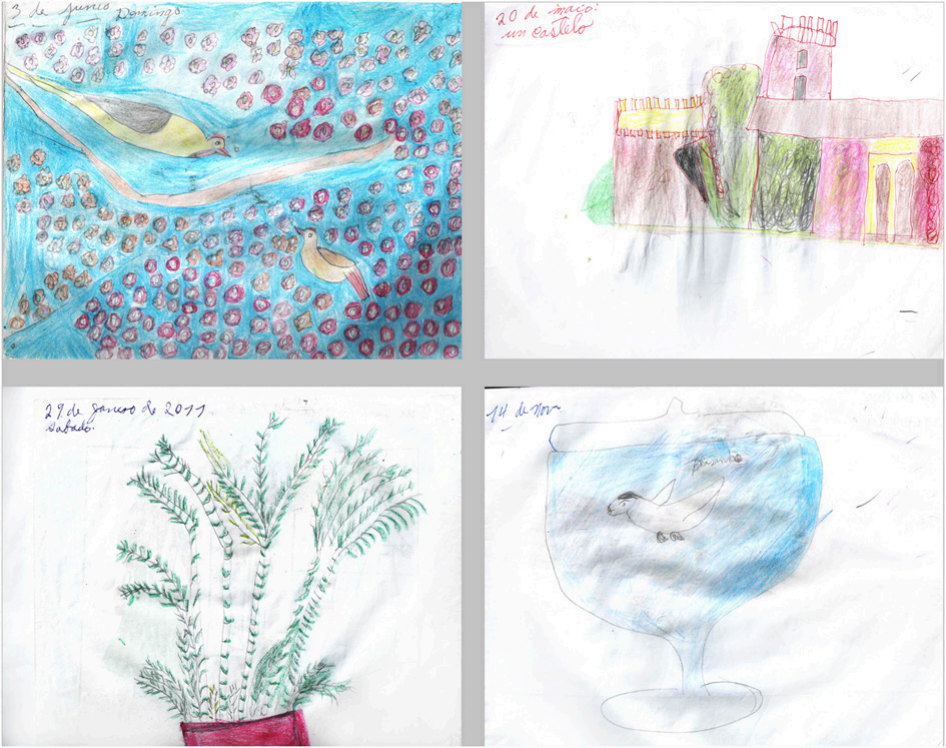
**Drawings with higher and lower CAT scores. Left panel:** drawings with the highest global scores (8.5 for the upper drawing, range 4–10; 7.4 for the lower drawing, range 3–10). **Right panel:** drawings with the lowest global scores (5.8 for the upper drawing, range 4–10; 6.0 for the lower drawing, range 2–10).

The patient was never notably interested in art. However, during the course of her disease, she began to draw compulsively on a daily basis (Figure 2). We sought to systematically analyze her drawing production using independent tools for this assessment. For this purpose, we used the consensual assessment technique (CAT; Amabile, 1982) to measure the global creativity of each drawing combined with a questionnaire adapted from Drago and colleagues (Drago et al., 2006a). The criteria assessed in this questionnaire included “Aesthetics”: How beautiful is the painting? “Closure”: How complete is the painting? “Abstraction”: How abstractive is the painting? “Obsessions/Repetition”: How obsessive/repetitive is the painting? “Evocative Impact”: How strongly does the painting induce feelings or thoughts? “Novelty”: How original or new is the painting? “Representation”: How well is the subject of the painting rendered? “Technique”: How much skill does the painting demonstrate?

We selected 20 drawings from May 2010 to September 2013 and asked 12 independent professional visual artists from Brazil (5 men, 7 women, aged from 31 to 70 years old, 5 of which were professors at Fine Art universities, most of which had formal artistic training in Fine Arts) to judge the drawings according to global creativity and the criteria explored in the questionnaire. The experts were also encouraged to make free comments. This expert group was blind to the clinical condition of the patient, and no information on her artistic status or training was given.

The results of this evaluation are presented in Table 5. The mean global creativity score across experts and drawings was 6.6, but varied markedly depending on the expert, ranging from 3.2 to 9.6. Scores for each criterion also showed a considerable heterogeneity between the experts ranging from 0 to 10 for each drawing. This heterogeneity suggests that the 12 scorers, all experts in the domain of visual arts, had a different conception of what creativity and its related features should be.

**Table 5:** **CAT assessment of the drawings from patient YCFZ (2010–2013)**.

	**Global Score**	**Aesthetics**	**Closure**	**Abstraction**	**Obsessions/repetitions**	**Evocative impact**	**Novelty**	**Representation**	**Technique**
Artist 1	4.8	4.4	5.7	4.1	4.1	3.8	3.6	4.7	3.3
Artist 2	3.2	0.3	9.2	1.5	8.5	0.3	0.9	3.0	0.0
Artist 3	3.2	0.3	9.2	1.5	8.5	0.3	0.9	3.0	0.0
Artist 4	9.4	9.1	9.4	9.1	9.1	9.8	9.3	9.8	9.6
Artist 5	6.6	4.6	4.4	1.5	3.4	4.0	2.1	3.9	3.6
Artist 6	9.6	9.5	10.0	0.0	0.0	8.7	10.0	9.8	10.0
Artist 7	9.3	8.9	9.2	1.3	5.3	8.2	8.4	8.7	8.8
Artist 8	3.2	3.0	3.1	3.3	4.6	3.6	3.2	3.0	2.9
Artist 9	7.0	5.6	7.7	3.9	4.5	6.2	6.5	7.5	6.5
Artist 10	7.9	7.2	8.5	7.5	3.7	7.6	7.8	7.3	6.5
Artist 11	6.8	5.7	8.4	1.7	5.4	6.1	3.8	7.4	5.6
Artist 12	8.5	8.2	9.3	5.1	4.9	6.8	7.8	9.5	8.7
**Mean**	**6.6**	**5.6**	**7.8**	**3.3**	**5.2**	**5.4**	**5.3**	**6.4**	**5.4**
**SD**	**2.5**	**3.2**	**2.2**	**2.7**	**2.5**	**3.1**	**3.3**	**2.8**	**3.5**
**Min**	**3.2**	**0.3**	**3.1**	**0.0**	**0.0**	**0.3**	**0.9**	**3.0**	**0.0**
**Max**	**9.6**	**9.5**	**10.0**	**9.1**	**9.1**	**9.8**	**10.0**	**9.8**	**10.0**

*The professional artists scored (from 0 to 10) each of the drawings for global creativity and according to the following criteria (adapted from Drago et al., 2006a): Aesthetics: How beautiful is the painting? Closure: How complete is the painting? Abstraction: How abstractive is the painting? Obsessions/Repetition: How obsessive/repetitive is the painting? Evocative Impact: How strongly does the painting induce feelings or thoughts? Novelty: How original or new is the painting? Representation: How well is the subject of the painting rendered? Technique: How much skill does the painting demonstrate? Mean scores attributed by each judge to the 20 assessed drawings are provided together with standard deviation, minimum and maximum values (in bold)*.

**Figure 3:**
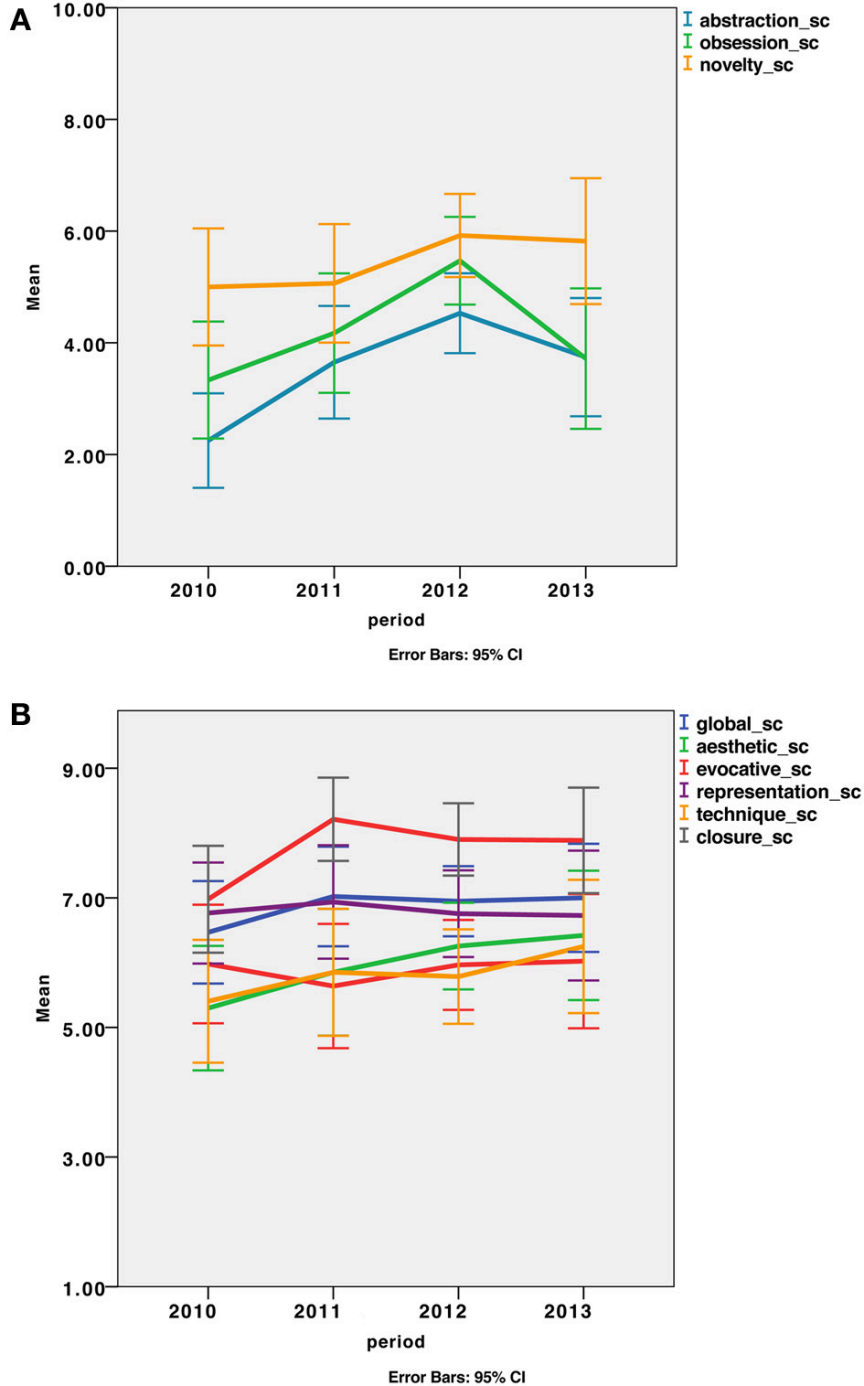
**Scores of the drawings across a 36-month period. (A)** Subscores with a significant improvement between the first (2010–2011) and the second period (2012–2013) of the drawings. Significant increases were observed for the abstraction, obsession and novelty subscores. **(B)** The global score and several subscores did not show a significant improvement between the first and the second period, though all scores increased.

CAT does not give an absolute assessment of creativity but provides relative scores enabling the comparison between different productions or different groups of participants. Therefore, we attempted to evaluate the evolving profile of the patient's drawings across time periods. First, we pooled drawings performed each year from 2010 to 2013 and looked at scores across the years (Figure 3). We observed an increase in scores from the first drawings (2010) to the last drawings (2013) in all of the evaluated aspects. Then, we statistically compared two periods: an early (drawings from 2010 and 2011; *n* = 8) and a late period (those from 2012 and 2013; *n* = 12) using a non-parametric Wilcoxon signed rank test. An increase in creativity scores was statistically significant for abstraction (*Z* = −2.756, *p* = 0.006), obsession (*Z* = −2.045, *p* = 0.041) and novelty (*Z* = −2.312, *p* = 0.021) subscores (Figure 3).

In their free comments, expert artists mentioned that most of the drawings were beautiful and creative, drawn with care, and found the compositions interesting or original. They insisted on the “naïve” character of the drawings, frequently describing them as simple and infantile (“these drawings are similar to those from my daughter of 6 years of age,” translated general comment from expert 1). The experts agreed on the representational rather than abstract nature of the productions. Repetitions, obsessions, or stereotypies were diversely interpreted. Many experts highlighted the repetitive and obsessive character of the drawings, but they often found them useful for the composition, the expression, or the rhythm of the picture, and gave low obsession scores for this reason. There was a large variability in the scores for repetitions and obsessions (minimal 0, maximal 9.1, with a mean of 5.2). The drawings were often described as expressive and containing negative emotions (“sinister paranoid atmosphere,” translated from expert 8 about drawing 19), but harmony was also evoked for some of them. Other comments highlighted bizarre or interesting compositions or strange/poor color choices.

Overall, the quantitative and qualitative creativity assessments showed great heterogeneity, especially in the general creativity of the drawings, the role of repetitions in the composition, or the emotional content. The disparity of judgment between the professional artists with academic training for most indicates that personal subjectivity strongly influenced the scoring. Despite a large inter-judge variability, an improvement of the patient's artistic skills was considered during a 3-year evolution period, especially for the abstraction, novelty, and repetition criteria, while language and autonomy declined. This suggests that the artistic creative capacity of the patient did not parallel her cognitive deterioration.

This observation is consistent with the potential emergence of an artistic inclination during the evolution of bvFTD, as previously reported, and highlights the interference between cognitive and behavioral frontal symptoms and creative production.

## Discussion: what do artistic patients tell us about creativity?

The difference between controlled patient studies and medical reports of creativity following frontal damage raises interesting questions regarding the mental components of creative thinking, their measurements, and their neural bases. Experimental approaches of creativity have demonstrated that various PFC regions are critical to creative capacity. Conversely, some frontal patients exhibit new or significant artistic productions despite their frontal dysfunctions, as was the case for the reported patient. Can this be explained? Does this mean that their creative capacities increased?

### Clinical considerations for patients with new or significant artistic production

Artistic facilitation is a rare phenomenon in neurological patients. The link between artistic production and neurological diseases appears to be anecdotal, especially when the high incidence of strokes and neurodegenerative diseases are considered. SD (FTD with temporal prominent atrophy) is the most frequent diagnosis associated with increased creative production (Table 2). In contrast with controlled studies that included unselected patients with neurodegenerative diseases, case reports point to particular patients who are especially concerned with making art. To our knowledge, no such exceptional patient with artistic facilitation has been explored using theory-based creativity tests. So it cannot be ruled out that controlled studies with unselected patients may miss some exceptional patients.

Because artistic facilitation has been observed in diseases as different as temporal and frontal variants of FTD, Alzheimer's disease, or stroke affecting distinct brain regions, clinical reports do not argue for a specific neuroanatomical or neuropsychological pattern associated with enhanced artistic production. For instance, it has been proposed that the emergence of artistic talent in FTD patients results from the release of the inhibition exerted by anterior regions over the posterior regions involved in visuospatial processing (Kapur, 1996; Mendez, 2004; Seeley et al., 2008). This theory may not explain the improved or preserved creative output in patients with predominant posterior injury (Annoni et al., 2005; Fornazzari, 2005) or in patients with no frontal dysfunction (Schrag and Trimble, 2001; Thomas-Anterion et al., 2010). Nevertheless, it is remarkable that most positive changes of artistic abilities concerned visual arts (41 cases out of 54 reviewed, including the current case report) when most patients had preserved visuospatial skills each time this was reported. In the related cases of creative production associated with either bvFTD or SD, degeneration mostly affected the left temporal and/or frontal regions, which may explain the predominance of visual arts in the patients' production being more related to the visuospatial functions of the right hemisphere. However, Wu and colleagues (Wu et al., 2013) recently reported two SD patients in whom the emergence of artistic activities in the verbal domain was associated with a predominantly left atrophy. Additionally, this left-right hypothesis is not in agreement with functional imaging data, as the meta-analysis from Gonen-Yaacovi and colleagues demonstrated a left dominance of activation in both verbal and visual tasks. Unfortunately, most of the published clinical reports do not provide extensive or detailed neuropsychological and anatomical data, which would enable a better characterization of the relationship between frontal or visuospatial alterations and creative output.

### Ties between frontal symptoms and artistic productions

**Figure 4:**
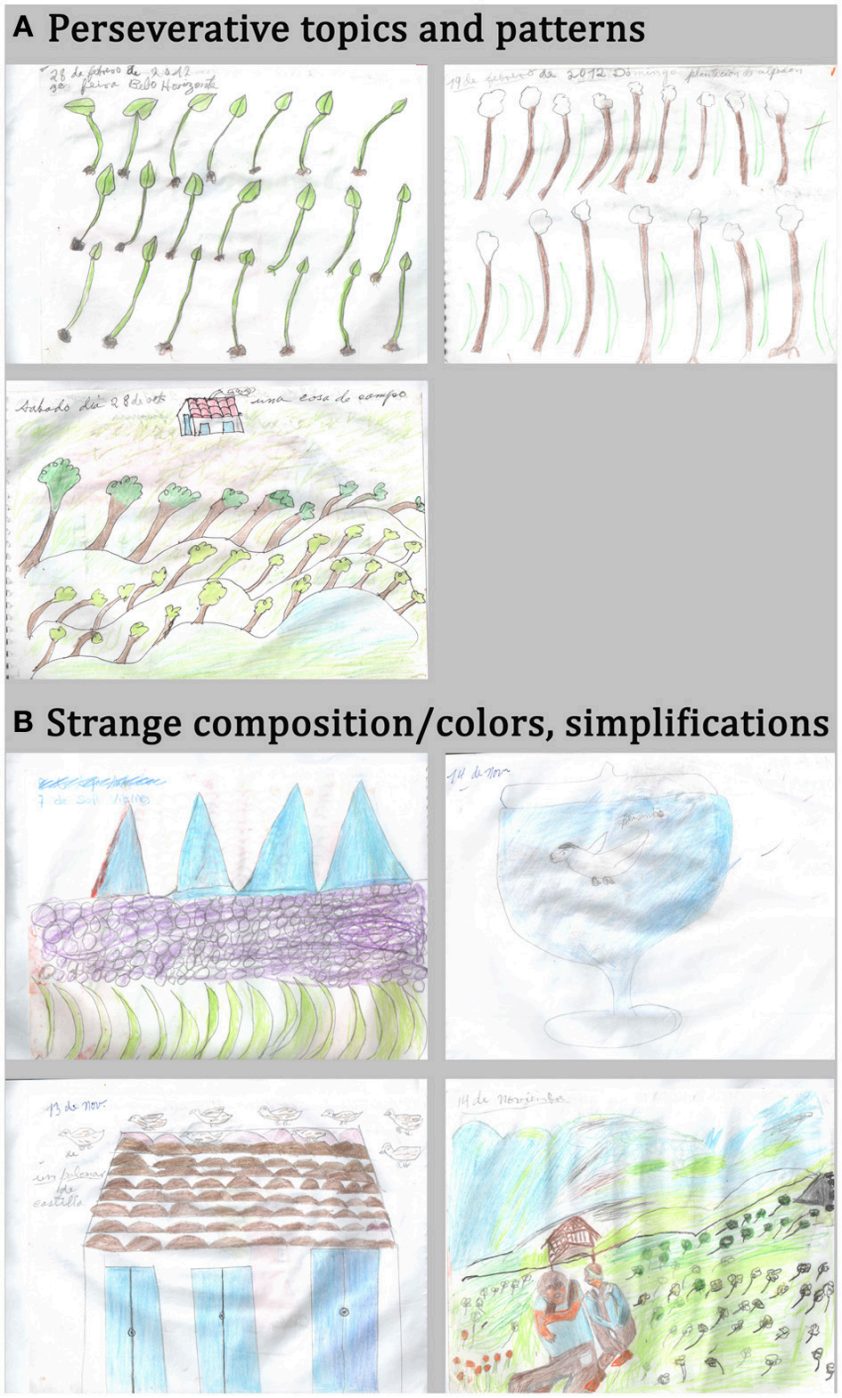
**Possible frontal manifestations expressed in the patient's drawings. (A)** Perseverative topics and patterns. **(B)** Strange composition, color choices and simplifications.

Some behavioral disorders associated with frontal damage may account for or parallel artistic expression, as suggested previously (Rankin et al., 2007; de Souza et al., 2010; Palmiero et al., 2012; Schott, 2012) and highlighted in our reported case. From a neurological point of view and based on the neuropsychological profile of our patient, we fist concluded that some frontal symptoms are possibly interfering with the drawings, while preserved visuospatial abilities enable their execution. The urge to draw on a daily basis and the huge amount of productions are possibly related to personality changes and compulsive behaviors provoked by frontal damage. Repetitive topics (plants, animals, people) and patterns (volcano, leaves) may be the manifestation of perseverations and stereotypies due to the frontal syndrome. Strange composition and infantile features may be explained by poor planning abilities (Figure 4).

Many patients with so called “artistic improvement” presented compulsive and/or obsessive behaviors (Finkelstein et al., 1991; Miller et al., 1998; Miller and Hou, 2004; Lythgoe et al., 2005; Serrano et al., 2005; Thomas-Anterion et al., 2010). As pointed by Schott (2012), in such patients, “a strong preference for a single art medium, a restricted focus on artistic themes, repetition, compulsion and seeking for perfection (…) enabled remarkable artistry to be achieved.” The patient we report on also produced drawings in a compulsive manner; this may partly account for the acquisition of an artistic technique. The fact that her last drawings received higher scores than the first drawings (produced 3 years prior) supports this assumption. Compulsive and/or obsessive behaviors are a major symptom of bvFTD (Rascovsky et al., 2011). These behaviors are surprisingly in contrast with the apathy also frequently observed in bvFTD, as well as with the cognitive inertia associated with a poor fluency, as was the case in our patient. Compulsive behaviors are usually associated with severe disorders of social conduct. For example, the patient reported by Miller et al. (1998) developed new photographic skills during the course of FTD. Pictures were taken compulsively to obtain a “perfect image.” However, at the same time, this compulsive demeanor also produced socially inappropriate behaviors, leading to severe social constraints, and ultimately to institutionalization. The patient we report also had ritualized behaviors that also led to social misconduct. In other words, the repetitive and ritualized behaviors related to frontal dysfunction may be expressed in the artistic domain, leading to new interests in making art or intense artistic activity with repetitive topics or productions. The reasons why some patients focus their compulsive behaviors on making art and others do not remain poorly understood.

Perseverations or patterning, which are also linked to frontal damage, were observed in our patient's drawings (trees and leaves, for instance). Surprisingly, our expert group remarked repetitions and made free comments about them but did not give especially high scores on the repetition criteria because they did not feel it was inappropriate or unaesthetic. A previous case-control study of creative production across bvFTD patients and normal controls (de Souza et al., 2010) showed that behavioral disorders, such as perseverations, may also partly explain the “originality” of some productions when frontal patients perform divergent thinking tests, but overall their originality score was impaired. Similarly, disinhibition, another cardinal symptom of frontal dysfunction, can interfere with creative activities, as also noted by de Souza et al. (2010); however this was not observed in the current case. Social disinhibition can lead to unexpected choices of topics, for instance with sexual content. The release of the inhibition exerted by frontal regions over the posterior regions may explain some unconventional or socially unusual aspects of creative productions as well as behaviors in frontal patients (Miller et al., 1996; Mell et al., 2003; Mendez, 2004; Miller and Hou, 2004; Drago et al., 2006b; Seeley et al., 2008).

In the cognitive sphere, some frontal lesions may help in overcoming knowledge constraints (Reverberi et al., 2005; Abraham, 2014). Patients with lateral prefrontal damage may experience a less sculpted (less constrained) response space in a given context than healthy subjects, enabling them to more easily consider any option, including those outside of contextual constraints (Reverberi et al., 2005). Overall, disinhibition or the loss of social conventions and associative knowledge may allow the emergence of creative productions (Miller et al., 1996, 2000; Miller and Hou, 2004; Liu et al., 2009). According to Rankin et al. (2007), productions from bvFTD patients may have an artistic value in the sense that they are freer from conventional representations and social conventions about art. It is more difficult to assume that this freeing from convention is an intentional and voluntary act.

Finally, our patient's drawings share other qualitative features that have been reported in previous FTD patients, especially with those described in Rankin and colleagues' study (Rankin et al., 2007) in which patient productions were assessed by scientists who had an interest in arts and not by professional artists. For instance, landscapes, people, animals and plants appear to be the preferred topics in frontal patient's productions. These preferred topics may be considered to be conventional and concrete but are often represented in an unusual way. The simplification of representations, judged as naïve or infantile, and unconventional or disordered composition with eccentricity of the subject, could be linked to a poor planning ability and lack of abstraction but could also contribute to the bizarreness and unusualness of the drawings.

Together, patient observations indicate that some clinical and behavioral symptoms of frontal dysfunction may facilitate the appearance of creative features in artistic products. This explanation cannot stand in the domain of creativity in which other frontal functions such as cognitive control, planning, mental manipulation, and abstraction are critical. Additionally, these observations raise the question of whether the artistic productions we observe reflect the same aspect of creative capacity and result from the same voluntary creative processes that are assessed in experimental creativity studies.

### Artistic and neuroscientific perspectives

Patient studies and clinical observations may highlight the probable difference between creativity evaluated from an artistic point of view and creativity evaluated from a neuroscientific perspective. In the field of Art, aspects such as emotional or evocative impact, provocation and message, aesthetic value, or technical mastery may be more important than in other domains such as sciences and technology. These aspects are not captured by the consensual definition of creativity that focuses on originality and appropriateness. Within the frame of this definition, a difference may also be noted: originality may often be considered to be a predominant condition for creativity in the artistic field in which appropriateness is difficult to apprehend; however, in other domains such as science, appropriateness is a requirement. For example, patients studied in de Souza et al. (2010) were often inappropriate in their responses, while no control subjects were. The sexual content of their drawings may be regarded as inappropriate in an experimental testing context but is usually well accepted in artistic works. This suggests that each domain of creative expression differently prioritizes originality and appropriateness and makes different demands on the mental operations to achieve them.

It is also important to mention that experimental and neuroimaging approaches do not assess motivational, conative, or emotional factors affecting creative drive. However, these factors appear to be important in real life creativity, such as in the spontaneous productions of patients with an artistic preoccupation. As highlighted by Schott (2012), these patients are often described “as obsessive about their art, with an urge to create.” It is then possible that emotional and motivational factors play an important role in real life creativity, including artistic creativity, but are poorly accounted for in experimental approaches of creativity. The latter are indeed based on cognitive theories of creative capacity with limited assessment of the emotional and conative aspects.

In our case, the score of evocative impact, which is intended to depend on the emotional expressiveness of the drawings, varied between the experts (0.3–9.8, with a mean value of 5.4). One method for analyzing the importance of emotional process in the artistic production of the patient would be to study the correlations between the scores on the CAT and objective measures of social-emotional cognition such as emotion recognition, empathy, and theory of mind. Unfortunately, these domains were not evaluated in our patient.

Finally, the difference between real life and experimental settings for measuring creativity is also in question. The evaluation of spontaneous patient productions was generally based on subjective assessments from authors, experts, or groups of judges. Our clinical case illustrates that subjective assessment, although framed by determined criteria and performed by experts in the field of visual arts, has a great inter-individual variability. Experimental theory-based approaches use more objective criteria to measure creative capacity, for instance fluency, flexibility, originality, or problem solving success. Several of these cognitive approaches have been used to study the neural basis of creativity in functional neuroimaging and in neurological patients. If theory-based approaches use more “objective” criteria, they are constrained by the hypothesis that they rely on. In other words, creativity tasks only assess the processes involved in creative capacity according to the cognitive model used. Each theory-based approach focuses on one or more aspects of the creative process, but none of them evaluates the creativity in all of its dimensions. In particular, theory-based creativity tasks do not necessarily capture artistic quality, even though they have been shown to be ecologically valid and statistically linked with artistic creativity (Kim, 2006; Plucker and Makel, 2010). On the contrary, theory-free creativity assessments, such as CAT, are not based on any particular theory of creativity, which means that their validity is not dependent upon the validity of any particular theory. Unfortunately, our patient was no longer able to perform experimental creativity tests at the time of the consultation; thus, we are not able to compare both approaches to creativity assessment in a case of artistic preoccupation.

Overall, the creativity attributed to patients preoccupied with arts during a frontal disease and creativity explored in experimental studies differ in several conceptual and experimental ways, and are probably affected differently by frontal symptoms.

## Overall, can hypofrontality facilitate creativity?

A common notion suggests that losing control, especially relaxing social and emotional inhibitions or conventions, may favor personal expression and creativity. The use of drugs such as alcohol may aim to approach this state. Several artistic streams are based on the spontaneous, non-controlled generation of ideas or objects, for instance using automatic writing or random painting. In the neuroscientific literature, some studies suggest that unconscious and uncontrolled processes facilitate divergent thinking and insight problem solving (Yaniv and Meyer, 1987; Dijksterhuis and Meurs, 2006; Dorfman et al., 2008; Zhong et al., 2008; Ritter and Dijksterhuis, 2014). Because control in behavioral, affective, social and cognitive spheres is largely associated with the functions of the PFC, the notion that hypofrontality could favor creativity may be valid. A recent theory (Chrysikou et al., 2013) also postulates that hypofrontality may enhance some aspects of creativity: the availability of bottom-up information that is usually filtered by the PFC may favor a breaking away from rule-based thinking. This is reinforced by the fact that some frontal patients appear to have abilities in some aspects of artistic expression.

The current review identified several clinical aspects of hypofrontality in the social, conative and cognitive domains that could explain some creative features of the patients' products. First, a social aspect related to the common view of hypofrontality is disinhibition. Social disinhibition can lead frontal patients to break with social conventions and propose unusual productions in creative (but also in uncreative) activities. Second, compulsive, repetitive or obsessive behaviors may lead to high productivity and improvements in technical skills. This obsessive-compulsive trait acts as a strong motivation toward a given activity. A third and more paradoxical aspect consists of a lower influence of habitual contextual associations in frontal patients. Patients with lateral PFC damage may be less constrained by learned rules, which may facilitate some problem-solving tasks (Reverberi et al., 2005). This aspect is paradoxical because it is in apparent opposition to the acknowledged role of the inferolateral PFC in overcoming prepotent responses [as observed in functional imaging and patient studies using Stroop tasks, no-go tasks or Hayling tasks (Aron et al., 2003; Brass et al., 2005; Picton et al., 2007; Volle et al., 2012), as well as in contextual control (Azuar et al., 2014)]. Overcoming prepotent responses and contextual control are both thought to play a role in creativity. Thus, whether highly creative people among the general population have more relaxed contextual constraints (as frontal patients may have) or increased abilities to intentionally overcome these constraints is an interesting topic for future research.

If hypofrontality generally evokes signs of disinhibition and poor control (usually associated with lateral and ventral portions of the PFC), we should also consider other roles of PFC in cognition that have been more recently highlighted. For instance, the medial PFC is part of the default network (Buckner et al., 2008), a set of functionally connected brain regions in which activity decreases when tasks require more focal attention, effort, or control. This network has been associated with spontaneous cognition and mind wandering (Gilbert et al., 2007; Mason et al., 2007; Christoff et al., 2009; Andrews-Hanna et al., 2010). This network can be distinguished from the set of regions functionally connected to lateral PFC (Gilbert et al., 2010). Some recent studies highlighted the role of the default network and of spontaneous cognition in creativity (Takeuchi et al., 2012; Wise and Braga, 2014). Medial PFC is also involved in semantic processing (Buckner et al., 2008; Binder et al., 2009) and in semantic aspects of creativity (Green et al., 2012; Abraham, 2014). These data suggest that the lateral PFC is engaged in rule-based thinking, while the medial PFC appears to be involved in a more spontaneous mode of thinking such as associative thinking. The rostral PFC may act as a switch (Burgess et al., 2007) between these two modes. Both thinking modes are thought to be required for creativity, as suggested in several models (Vartanian et al., 2007; Gabora, 2010; Ward and Kolomyts, 2010; Ellamil et al., 2012). For instance, the uncontrolled association of ideas triggered by perceptual or emotional stimuli may favor unusual responses but may also lead to inappropriate responses if the control mode does not filter. How each mode is affected by frontal lesions and how it impacts creative capacity is poorly known. The consequences of damage to the rostromedial PFC on creativity may lead to poorer originality (de Souza et al., 2010; Shamay-Tsoory et al., 2011), but the mechanisms of this change and its relationship with the default network functions are unexplored.

Overall, the classical view in which PFC supports top-down controlled processing while subcortical and posterior brain regions are engaged in bottom-up uncontrolled processing may be more balanced regarding creativity. If the lateral PFC is largely associated with top-down control, some other PFC regions may be involved in the uncontrolled or bottom-up processing that is spontaneous cognition, including semantic associations and mind wandering. The interaction between controlled and spontaneous cognition *via* connectivity between the lateral and medial PFC networks (Spreng et al., 2010) may enable both original and appropriate ideas to emerge.

## Conclusion

The functional and anatomical organization of the PFC supports different aspects of behavioral adaptation in humans, suggesting its role in the adaptive aspects of creativity as they are emphasized in its definition (i.e., creating something original and appropriate). Functional neuroimaging and experimental patient studies also suggest that the PFC, in particular the anterior PFC, may also play a critical role in originality aspects of creativity. Damage to the PFC may alter the intentional appropriateness and originality of patient productions by altering planning, fluency, mental flexibility, rule-based thinking, or abstraction. However, clinical observations of frontal damage patients suggest that some symptoms associated with frontal damage provoke cognitive, conative, and behavioral changes, including social disinhibition, compulsive behaviors, emotional distortions, and the relaxing of cognitive constraints, which can motivate and favor artistic productions. However, artistic production is not synonymous with creativity, because creativity refers to aspects such as emotional expression, evocative impact, aesthetic, and technical abilities, which are present in art but not necessarily in other domains of creativity. Art is thus difficult to capture using theory-based creativity tasks, and to our knowledge, patients with facilitation in the artistic domain have not been tested experimentally with such tasks. Therefore, whether these rare frontal patients increase their real creative capacity does not have a yes or no answer. Using theory-based creativity tasks, functional imaging and patient data suggest that distinct PFC subregions differently affect the different aspects of creativity. PFC cannot be considered as a unitary structure, and exploring its organization and interactions subserving different creativity processes, including controlled and spontaneous cognition, as well as social and affective aspects, may provide a more precise answer.

### Conflict of interest statement

The authors declare that the research was conducted in the absence of any commercial or financial relationships that could be construed as a potential conflict of interest.
